# Microfluidic device to attain high spatial and temporal control of oxygen

**DOI:** 10.1371/journal.pone.0209574

**Published:** 2018-12-20

**Authors:** Sandra F. Lam, Venktesh S. Shirure, Yunli E. Chu, Alan G. Soetikno, Steven C. George

**Affiliations:** 1 Department of Biomedical Engineering, Washington University in St. Louis, St. Louis, Missouri, United States of America; 2 Department of Biomedical Engineering, University of California Davis, Davis, California, United States of America; Texas A&M University College Station, UNITED STATES

## Abstract

Microfluidic devices have been successfully used to recreate *in vitro* biological microenvironments, including disease states. However, one constant issue for replicating microenvironments is that atmospheric oxygen concentration (21% O_2_) does not mimic physiological values (often around 5% O_2_). We have created a microfluidic device that can control both the spatial and temporal variations in oxygen tensions that are characteristic of *in vivo* biology. Additionally, since the microcirculation is responsive to hypoxia, we used a 3D sprouting angiogenesis assay to confirm the biological relevance of the microfluidic platform. Our device consists of three parallel connected tissue chambers and an oxygen scavenger channel placed adjacent to these tissue chambers. Experimentally measured oxygen maps were constructed using phosphorescent lifetime imaging microscopy and compared with values from a computational model. The central chamber was loaded with endothelial and fibroblast cells to form a 3D vascular network. Four to six days later, fibroblasts were loaded into the side chambers, and a day later the oxygen scavenger (sodium sulfite) was flowed through the adjacent channel to induce a spatial and temporal oxygen gradient. Our results demonstrate that both constant chronic and intermittent hypoxia can bias vessel growth, with constant chronic hypoxia showing higher degrees of biased angiogenesis. Our simple design provides consistent control of spatial and temporal oxygen gradients in the tissue microenvironment and can be used to investigate important oxygen-dependent biological processes in conditions such as cancer and ischemic heart disease.

## Introduction

Although hypoxia, or low oxygen tension, is a central feature of many different diseases, it is also a part of normal physiological states [1–3]. Hypoxia can be chronic (sustained oxygen tension < 5% O_2_ for > 24 hours), as in the case of ischemic heart disease and wound healing, or intermittent (repeated oxygen tension < 5% O_2_ for < 24 hours) as in the case of some tumors, sleep apnea, and exercise [4–7]. Moreover, diseases such as cancer can exhibit both chronic and intermittent hypoxia states which can affect different processes of tumor progression. The rapid rate of diffusion of abundant oxygen in gas and liquid systems has made it difficult to experimentally study the impact of hypoxia at small spatial scales, in particular, to replicate *in vivo* intermittent hypoxia conditions. The presence of atmospheric air (~ 160 mmHg or 21% O_2_) provides a regular source of “contamination” as physioxia or physiological oxygen concentration for tissues are on average about 38 mmHg or 5% O_2_ [8]. Although previous literature often compares hypoxic conditions to normoxic conditions, we chose to compare hypoxic conditions to physioxia (5% O_2_) conditions because it is more physiologically relevant. To perform these studies, even though the incubator oxygen tension may be set to 5% O_2_, controlling the spatial distribution of oxygen within the cell culture is not possible. As the tissue expands farther away from the vessel network, an oxygen gradient is established, with the cells farthest away experiencing the lowest oxygen tension. In addition, it has been shown that gradients are important for chemical and mechanical factors for recapitulating the physiological microenvironment [9,10].

Here, we present a microfluidic device that can precisely control oxygen tension over spatial and temporal dimensions on the order of microns and minutes, respectively. This level of control permits the interrogation of physiologically relevant hypoxia in a wide range of normal and diseased tissues. Our strategy leverages the small dimensions of microfluidic devices that minimize the distance of diffusion for oxygen to control precise gradients. Microfluidic systems provide faster oxygen cycle switching than some commercial systems, (e.g., Eppendorf Galazy 48R), which can take up to 30 minutes to equilibrate to a new oxygen concentration. In addition, previous methods of studying hypoxia in microfluidic devices often involve nearby channels to flow nitrogen gas to create an oxygen sink [11–13]. While this can create controlled oxygen gradients, the need for nitrogen tanks is cumbersome. Furthermore, the use of a compressed gas can introduce bubbles and pervaporation in the culture chambers, which can compromise the experimental conditions.

Our approach utilizes a simple and inexpensive aqueous solution of sodium sulfite as an oxygen scavenger. This has several advantages over previous methods of controlling oxygen: 1) our method reduces pervaporation and avoids introduction of gas bubbles in long term cell cultures; 2) our method avoids exposure of cells to non-physiologic oxygen scavenging chemicals; 3) our method is cost effective as expensive gas tanks, gas mixers, or oxygen scavengers are not required; 4) our method offers more leverage to precisely control oxygen gradients as kinetic properties (e. g. concentration of sodium sulfite) in addition to mass transport (e. g. flow rate) can be finely regulated to control the gradients. This easily prepared solution is flowed in a fluidic channel running parallel to the tissue chambers. Because this channel is not connected to the tissue chambers the oxygen scavenger will not produce any unwanted side effects to the tissues. Moreover, most *in vitro* studies mimic intermittent hypoxia cycle between 20% and 1% O_2_ to ensure two different oxygen states [14–16]. However, 20% O_2_ is representative of hyperoxia and does not accurately represent *in vivo* intermittent hypoxia. Subsequently, because we can measure the spatial and temporal oxygen distribution within our device, we can easily switch between the more physiological concentration of 5% and 1% O_2_. Thus, we believe that our results more accurately represent physiological response to intermittent hypoxia. Furthermore, although previous studies have shown the effects of using an oxygen scavenger in a 20% O_2_ incubator, the range of oxygen concentrations often go far above physiological values [17].

To demonstrate the biological relevance of our microfluidic system, we used an *in vitro* model of angiogenesis. During hypoxia, new blood vessels can sprout from existing vessels (angiogenesis) as a normal biological response to relieve hypoxia by increasing blood flow and delivering oxygen. On the other hand, hypoxia can also lead to necrosis of the tissue if there is a failure to encourage new vessel growth. These two drastically different outcomes are both possible during hypoxia because both pro-angiogenic and anti-angiogenic genes can be activated, and this balance dictates the outcome [18]. Thus, it is important to determine the subtleties of spatial and temporal variations in oxygen and how this can bias vessel growth. Our theoretical and experimental results demonstrate spatial and temporal control over the oxygen tension within a microfluidic platform. In addition, the simple and flexible design will prove useful for a range of additional biological applications. In this study we show the effects of physioxia (P), constant chronic hypoxia (CH), and intermittent hypoxia (IH) on true 3D angiogenesis.

## Materials and methods

### Design and microfabrication of microfluidic device

**Fig 1:**
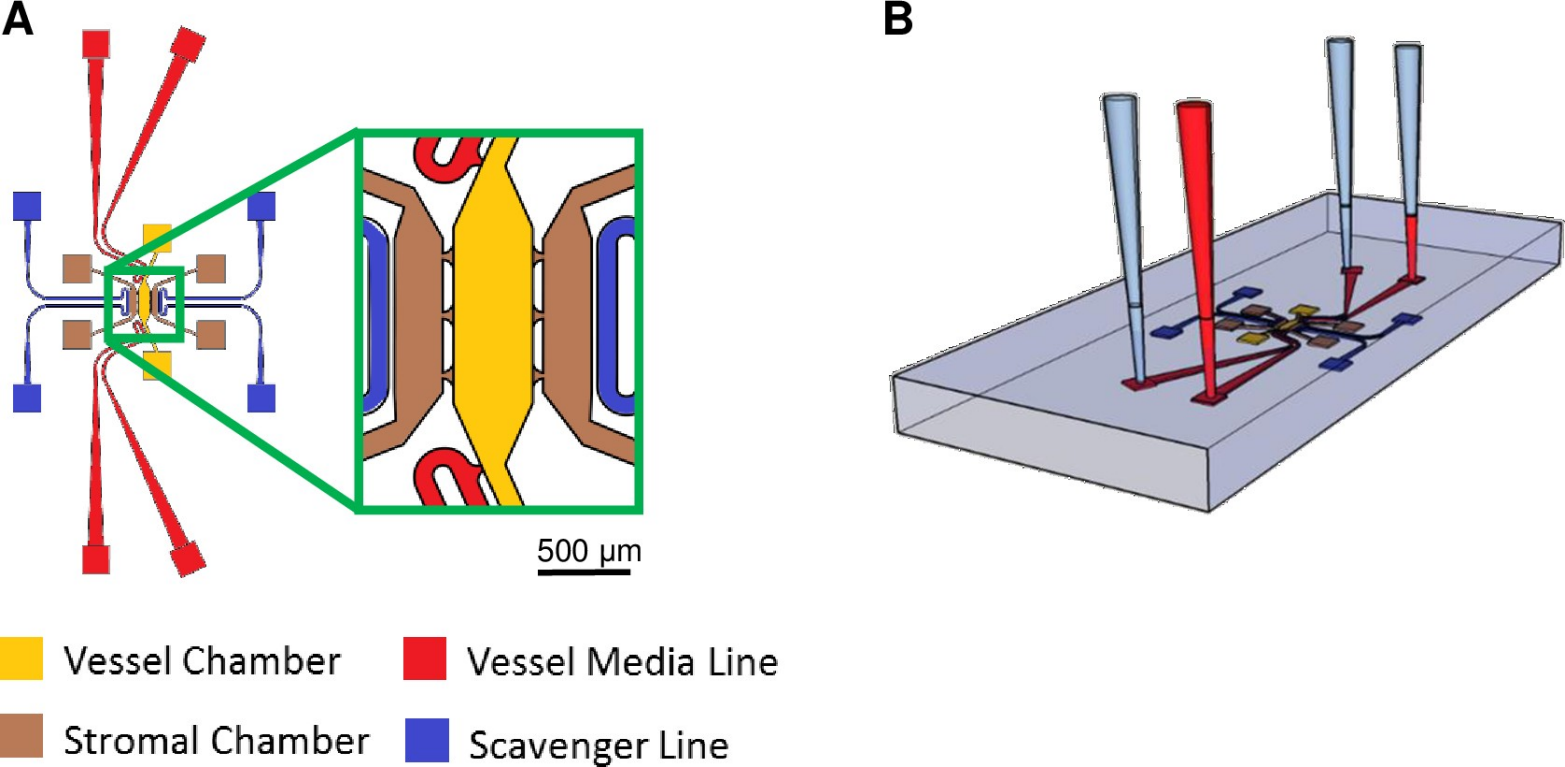
Microfluidic device schematic. (A) The design of the microfluidic device with the central vascular chamber (yellow) and adjacent stromal chambers (brown). Scavenger channels (blue) are placed next to the stromal chambers and media lines (red) feed the vascular chamber. (B) Experimental setup for the microfluidic devices. A difference in hydrostatic pressure head between the inlet and outlet of each microfluidic line creates a drop in pressure between the two sides of the vascular chamber to induce convective interstitial flow throughout the device.

The microfluidic device design has three parallel chambers connected through capillary burst valves measuring 30 μm wide and 100 μm high to facilitate communication between chambers (Fig 1A). The central compartment (vascular chamber) is the largest with a volume of 0.06 mm^3^ (1.5 mm x 0.4 mm x 0.1 mm), and the two adjacent chambers are smaller with a volume of 0.02 mm^3^ (0.8 mm x 0.25 mm x 0.1 mm). Only the central compartment has media lines that are connected through capillary burst valves on each end. This design allows for separate chambers to contain different compositions of cells and extracellular matrix as well as to be loaded into the device at different time points. The two media lines were designed to control the flow of media via a difference in hydrostatic pressure head between the two lines (Fig 1B). The flow was controlled to mimic physiological fluid velocities of approximately 1–10 μm s^-1^. Lastly, microfluidic channels adjacent to each of the smaller tissue chambers are present and are used to deliver the oxygen scavenger to create the oxygen gradient.

Once a computer-aided design (CAD) of the device was created, the design was printed and standard photolithography methods were used to build the device. Briefly, a negative photoresist, SU-8 3050, (MicroChem, Newton, MA) was spun onto a silicon wafer to a height of 100 μm. Ultraviolet light exposure crosslinked the pattern of the device into the photoresist. After the pattern on the wafer was developed, the mold was silanized and polydimethylsiloxane (PDMS; Dow Corning, Elizabethtown, KY) was poured over the mold at a mixture of 10:1 (w/w) polymer to curing agent. The PDMS was cured in a 65°C oven overnight. The PDMS was then peeled off the mold and the inlet and outlet holes were punched into the device using an 18 gauge needle for the tissue chambers and 16 gauge needle for the media and scavenger lines. To bond the PDMS device to a glass slide, both pieces were first cleaned to remove debris and then plasma treated for 1 minute at 250 mTorr. After plasma treatment, the PDMS device was sealed to the glass slide and placed in a 120°C oven for a minimum of 15 minutes. Lastly, the device was sterilized with ultraviolet light before experimentation.

### Numerical simulations

To enhance our understanding of interstitial flow and oxygen concentrations within the device, we created a computational model of mass and momentum transport using COMSOL Multiphysics 5.2a (Burlington, MA) combined with the CAD model of the device. To simulate the flow throughout the device, a 3D steady state solution of the incompressible Navier-Stokes equations was calculated using no slip boundary conditions for all the walls. Properties of the components of the device are listed in Table 1 and are consistent with previous work. The *transport of diluted species* module was used to model the oxygen gradients throughout the device. Initial conditions for oxygen concentrations was set to 5% O_2_. Using Henry’s law, the outer boundaries of the device (PDMS-air interface) were set using the incubator level of 5% O_2_, and a Michaelis-Menten kinetic equation was used to simulate cellular metabolism of oxygen [19]. The PDMS-media interface was modeled using a pointwise constraint at the boundary in accordance with the stiff-spring method [20]. A media/PDMS partition coefficient of 0.18 was used in the model. The consumption of oxygen through the reaction with sodium sulfite (Na_2_SO_3_) was used from previously reported findings in the literature [19,21–26]. In brief, 1 mole of oxygen (O_2_) reacts with 2 moles of sodium sulfite (Na_2_SO_3_) to produce 2 moles of sodium sulfate (Na_2_SO_4_) with a reaction rate constant, k (Table 1):

$${2\mathrm{Na}}_{2}{\mathrm{SO}}_{3}{+O}_{2}\overset{k}{\to }{2\mathrm{Na}}_{2}{\mathrm{SO}}_{4}$$ (Eq 1)

**Table 1:** List of model parameters.

Parameter	Value	Unit	Source
Inlet pressure (high side)	25	mmH_2_O	-
Outlet pressure (high side)	0	mmH_2_O	-
Inlet pressure (low side)	10	mmH_2_O	-
Outlet pressure (low side)	0	mmH_2_O	-
Fibrin permeability	1.5E-13	m^2^	23
Diffusion of oxygen through PDMS	3.55E-9	m^2^ s^-1^	26
Diffusion of oxygen through water	2.1E-9	m^2^ s^-1^	20
Max rate of oxygen metabolism by cells (V_max_)	1.3E-17	mol s^-1^	19
Oxygen consumption at half of V_max_ (K_M_)	.008	mol m^-3^	19
Reaction rate constant (k)	5.77E-5	M^0.35^ s^-1^	25
Partition coefficient	0.18	-	20

Opposite the oxygen scavenging channel is a channel open to the atmosphere of the incubator, and thus set to 5% O_2_. The computational mesh of the model consists of 700,000–1,000,000 tetrahedral grid elements. For the intermittent hypoxia simulations, time dependent simulations were used with one hour cycles of flowing and stopping the reaction with sodium sulfite. To demonstrate the ability of the device to manipulate the gradient and oxygen tension, additional conditions were modeled to create several unique oxygen profiles. The simulated conditions (Table 2) include modifying the constant chronic hypoxia condition (CH) to having a reduced mass flow rate of the scavenger in the device (rCH), increased wall thickness between the scavenger channel and tissue chambers (wCH), and two scavenging lines (2CH). These modifications were added to demonstrate the versatility of oxygen gradients available with our device.

**Table 2:** List of experimental conditions.

Condition	Mass Flow Rate (mol s^-1^)	Wall Thickness (μm)	Number of Active Scavenger Lines	Frequency (h^-1^)	Time during cycle (min)
P	0	-	0	-	Constant
IH_on_	2 x 10^−6^	30	1	0.5	60
IH_off_	2 x 10^−6^	30	1	0.5	60
CH	0.7 x 10^−6^	30	1	-	Constant
rCH	0.07 x 10^−6^	30	1	-	Constant
wCH	0.7 x 10^−6^	60	1	-	Constant
2CH	0.7 x 10^−6^	30	2	-	Constant

P: Physioxia; IH_on_: Intermittent hypoxia while 2 x 10^−6^ mol s^-1^ Na_2_SO_3_ is flowing for an hour; IHo_ff_: Intermittent hypoxia while scavenger stopped flowing for an hour; CH: Constant chronic hypoxia of flowing 0.7 x 10^−6^ mol s^-1^ Na_2_SO_3_; rCH: Constant reduced mass flow rate of oxygen scavenger flowing at 1/10^th^ the speed of CH; wCH: Constant hypoxia with increased thickness of the PDMS wall between the scavenging channel and tissue chamber; 2CH: Constant hypoxia with two active scavenger line

### Oxygen measurements

To validate the oxygen concentration predictions from the computational model, we used phosphorescent lifetime imaging microscopy (PhLIM) that utilizes an oxygen sensitive dye, Oxyphor G4 (Oxygen Enterprises, Philadelphia, PA) [27,28]. This Pd-tetrabenzoporphyrin based dye is based on phosphorescence quenching, and we have already demonstrated its proficiency in an *in vivo* model [29]. The advantage of using the PhLIM technique with Oxyphor G4 is that experimental measurements at high spatial and temporal resolution can be achieved. To excite the phosphorescent dye, we used a 635 nm laser to modulate at 1 kHz with 5% duty cycle (FV1200 Olympus confocal with ISS (Urbana-Champagne, IL) phosphorescent lifetime instrumentation upgrade). The emission beam was collected through a miniTDU that was equipped with two Hamamatsu 7422p-50 detectors, which was coupled directly to the confocal head. To perform the oxygen measurement, the dye was added to the media of the device at a concentration of 20 μM after the end of the cellular experiment. The dye was allowed to equilibrate for at least an hour through the fibrin and tissue in the device. Oxygen measurements were made over the entire area of the three chambers for each of the three experimental conditions at room temperature. The phosphorescent lifetime of each pixel was calculated and was then converted into oxygen tension using a previously determined calibration curve at 22 ^o^C [27]. Because the oxygen measurements were performed at a different temperature than the cellular experiments, we quantified the reaction rate of sodium sulfite at both 22°C and 37°C to confirm that the reaction rate did not significantly depend on temperature over this temperature range (S1 Fig). For this experiment, 100 μl of sodium sulfite at various concentrations were prepared in a 96 well plate and oxygen measurements of the solutions were made at t = 60 s and from 50 μm above the bottom of the plate. The rate of change of oxygen was calculated by assuming that the solution was at 21% O_2_ concentration at t = 0 s and drops linearly until t = 60 s. At various sodium sulfite concentrations, the rate of consumption of oxygen does not significantly vary with temperature as tested by two factor ANOVA without replications.

### Cell culture

As previously described by our lab [30,31], endothelial colony forming cell-derived endothelial cells (ECFC-EC) were derived from cord blood and seeded on 1% gelatin-coated (Sigma-Aldrich, St. Louis, MO) flasks and provided with endothelial growth medium-2 (EGM-2; Lonza, Wakersfield, MD). Human umbilical cord blood was obtained following an approved protocol from the Washington University Institutional Review Board under the Human Research Protection Office. Normal human lung fibroblasts (NHLF) were commercially purchased and grown in fibroblast growth media (FGM-2, Lonza) before use in the experiments. All cells were cultured in a humidified incubator at 37°C, 5% CO_2_, and 20% O_2_ before loaded into the microfluidic device.

To study angiogenesis in the presence of spatial oxygen gradients, transduced ECFC-ECs that constitutively express fluorescence were used to continuously monitor growth of new blood vessels. To transduce the cells, we used a lentiviral particle titer from a human embryonic kidney primary cell line (HEK293T Cells, ATCC, Manassas, VA). The HEK293T cells were seeded in a 6 well plate at a density of 5.0 x 10^5^ cells/well and incubated for 24 hours in Dulbecco’s Modified Eagle Medium (DMEM, ThermoFisher, Waltham, MA) containing 10% fetal bovine serum (FBS, Sigma-Aldrich, St. Louis, MO) and no antibiotics. A solution of 250 μL of Opti MEM (Invitrogen, Carlsbad, CA), 7.5 μL of Lipofectamine 2000, and 3μg of plasmid DNA (1.5 μg pLVX-Azurite, .75 μg pMDLg/pRRE, .3 μg pRSV-Rev, and .45 μg pMD2.G) was mixed and incubated for 25 minutes at room temperature. Then, 500 μL of the solution was added dropwise to the wells of the HEK293T cells. The contents of each well was replaced with fresh media 24 hours later. After 48 hours of incubation, the viral supernantant was collected and spun down to exclude cell debris and stored at -80°C. A T-150 flask of ECFC-ECs were seeded and grown until about 30–40% confluency. Then, a 25 mL solution of EGM containing 8 μg/mL polybrene (Santa Cruz Biotechnology, Dallas, TX) and 500 μL of viral titer was added to the cells and allowed to incubate for 24 hours at 37°C. The contents of the flask were then aspirated and fresh EGM was added. Transduction efficiency was measured to be more than 90%.

### Device experimentation

For the vascular chamber, ECFC-ECs and NHLFs were trypsinized and resuspended in 16 mg mL^-1^ bovine fibrinogen (Sigma-Aldrich, St. Louis, MO) dissolved in Dulbecco’s Phosphate Buffered Saline (DPBS; ThermoFisher). The ECFC-ECs were collected at 5 x 10^6^ cells mL^-1^ and the NHLFs were collected at 10 x 10^6^ cells mL^-1^. Thrombin (Sigma-Aldrich) was prepared to a concentration of 50 U mL^-1^ in DPBS and added to the cell-fibrinogen solution. This initiated the polymerization process, and the solution was quickly pipetted into the central or vascular chamber of the device. After incubating the gel for 30 minutes at 37°C, EGM-2 was added to the media lines for the first 24 hours of the experiment and flow was maintained using a hydrostatic pressure head. Afterwards, the devices were placed in a 37°C, 5% CO_2_, and 5% O_2_ incubator and EGM-2 without growth factors was used to feed the tissue. The direction of the pressure gradient throughout the device was switched every day to ensure spatially homogenous growth [32,33].

Following the first four days of the experiment, vascular structures were apparent, and the adjacent chambers were then loaded with fibroblasts in a fibrin gel at a concentration of 7.5 x 10^6^ cells mL^-1^ using the previously described method. After 24 hours, the oxygen scavenger, sodium sulfite (Sigma Aldrich), was dissolved in deionized water and a 20 CC syringe was filled with the solution. The syringe was connected to Tygon tubing (Saint-Gobain, Valley Forge, PA) and the other end of the tubing was inserted into the scavenger channels of the device. The device was placed in the incubator such that at least 2 feet of the tubing remained inside the incubator. The solution was pushed through the left scavenger channel of the device using a syringe pump stationed outside the incubator. Only one of the scavenging channels had an oxygen scavenger flowing through it at specified times. The other channel (farthest right channel) was kept open to the incubator environment maintained at physioxia. To ensure that the two hypoxia conditions had the same average oxygen concentration over the duration of the experiment, 0.35 M sodium sulfite was flowed at 120 μl min^-1^ (mass flow rate of 0.7 x 10^−6^ mol s^-1^) continuously for the CH condition while 1 M flowed at 120 μl min^-1^ (mass flow rate of 2 x 10^−6^ mol s^-1^) was used for the IH condition with the pump programed for 1 hour of scavenger flow (IH_on_) and 1 hour of no flow (IH_off_). To mimic CH and IH, sodium sulfite was introduced through the left side of scavenger line for 6 more days until the end of the duration of the experiment. Because sodium sulfite constantly reacts with oxygen, it was important to maintain a constant flow of oxygen scavenger.

### Quantification of biased angiogenesis

At the end of the experiment, images were taken of the fluorescent vascular network in each of the chambers. To quantify sprouting angiogenesis, images were cropped to only contain the left or right stromal chamber. The images were randomized and blinded for image analysis in ImageJ. Multiple devices were used for each condition of P (n = 8), CH (n = 11), and IH (n = 17). We calculated biased angiogenesis using the following relationship:

$$\mathrm{Biased}\;\mathrm{Angiogenesis}=\frac{\mathrm{Vessel}\;\mathrm{area}\;\mathrm{in}\;\mathrm{left}\;\mathrm{or}\;\mathrm{right}\;\mathrm{chamber}}{\mathrm{Vessel}\;\mathrm{area}\;\mathrm{in}\;\mathrm{left}\;\mathrm{chamber}+\mathrm{vessel}\;\mathrm{area}\;\mathrm{in}\;\mathrm{right}\;\mathrm{chamber}}$$ (Eq 2)

In other words, the vessel area in each side chamber is expressed as the fraction of total angiogenesis into both chambers. A value of 0.5 indicates unbiased angiogenesis.

### Statistical analysis

Because the data was not normally distributed, the parameters are presented with medians and ranges from the 25% to 75% percentile. To determine if the biased angiogenesis and vessel area in the left chamber was significantly different compared to the right chamber, a nonparametric Mann-Whitney test was applied using GraphPad Prism. Significance was determined at the p < 0.05 level.

## Results

### Microfluidic device: Pressure and flow

**Fig 2:**
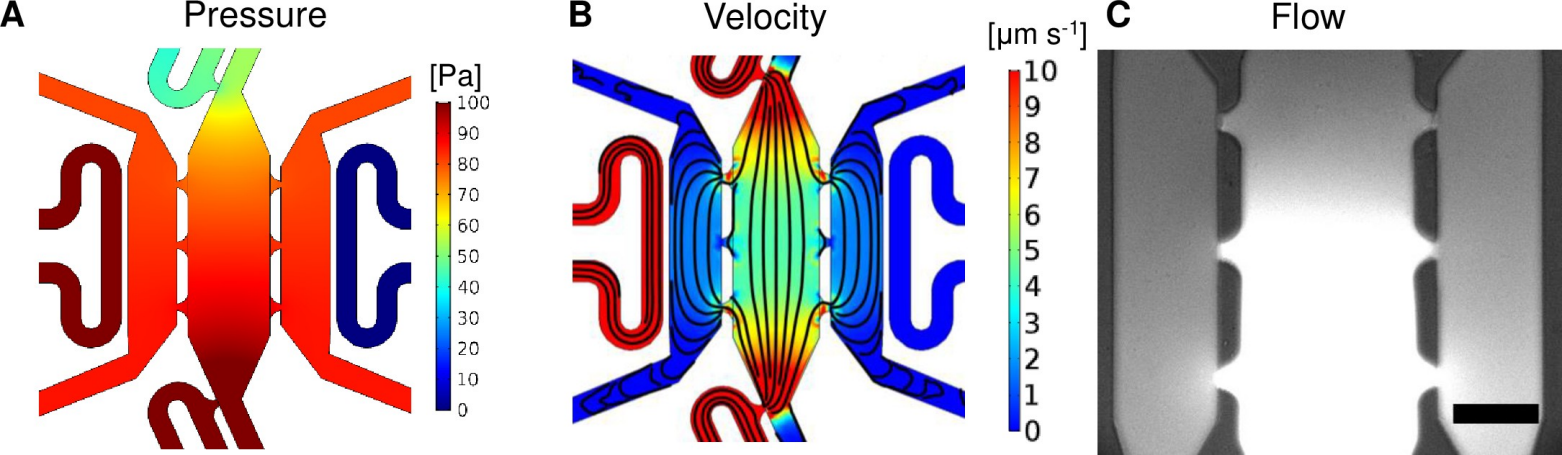
Microfluidic device characterization. (A) Surface map of the steady pressure (Pa) distributions inside the microfluidic device. (B) Surface map of the fluid velocity (μm s^-1^) and streamlines. (C) FITC-dextran was introduced through the media lines to demonstrate the direction of flow through the device (bottom to top and central chamber to outside chambers). The image was taken 30 minutes after introducing the dye. Scale bar = 200 μm.

After successful construction of the microfluidic device, we demonstrated desired pressure and velocity profiles (Fig 2A and 2B). The steady pressure profile of the device (Fig 2A) demonstrates a drop in pressure in the vascular chamber to the stromal chambers from 1 x 10^−2^ mmH_2_O to 0.5 x 10^−2^ mmH_2_O (approximately 9.8 x 10^−2^ to 4.9 x 10^−2^ Pa), respectively, with the stromal chamber averaging 0.85 x 10^−2^ mmH_2_O (8.3 x 10^−2^ Pa). Additionally, the device was designed to create physiologically relevant fluid velocities ranging between 1–10 um s^-1^ by appropriately adjusting the hydrostatic pressure head (25 mmH_2_O, Fig 2B). Another design consideration to note is that the adjacent stromal chambers do not have separate media lines; the chambers receive nutrients through the vascular chamber as shown by the streamlines (Fig 2B). To demonstrate this effect in the device, we loaded a microfluidic device with plain fibrin and introduced fluorescein isothiocyanate-dextran (FITC-dextran) through the media lines. The FITC-dextran shows the direction of fluid flow through the connecting pores and how media is able to reach all three tissue chambers (Fig 2C).

### Microfluidic device: Oxygen concentration profiles

**Fig 3:**
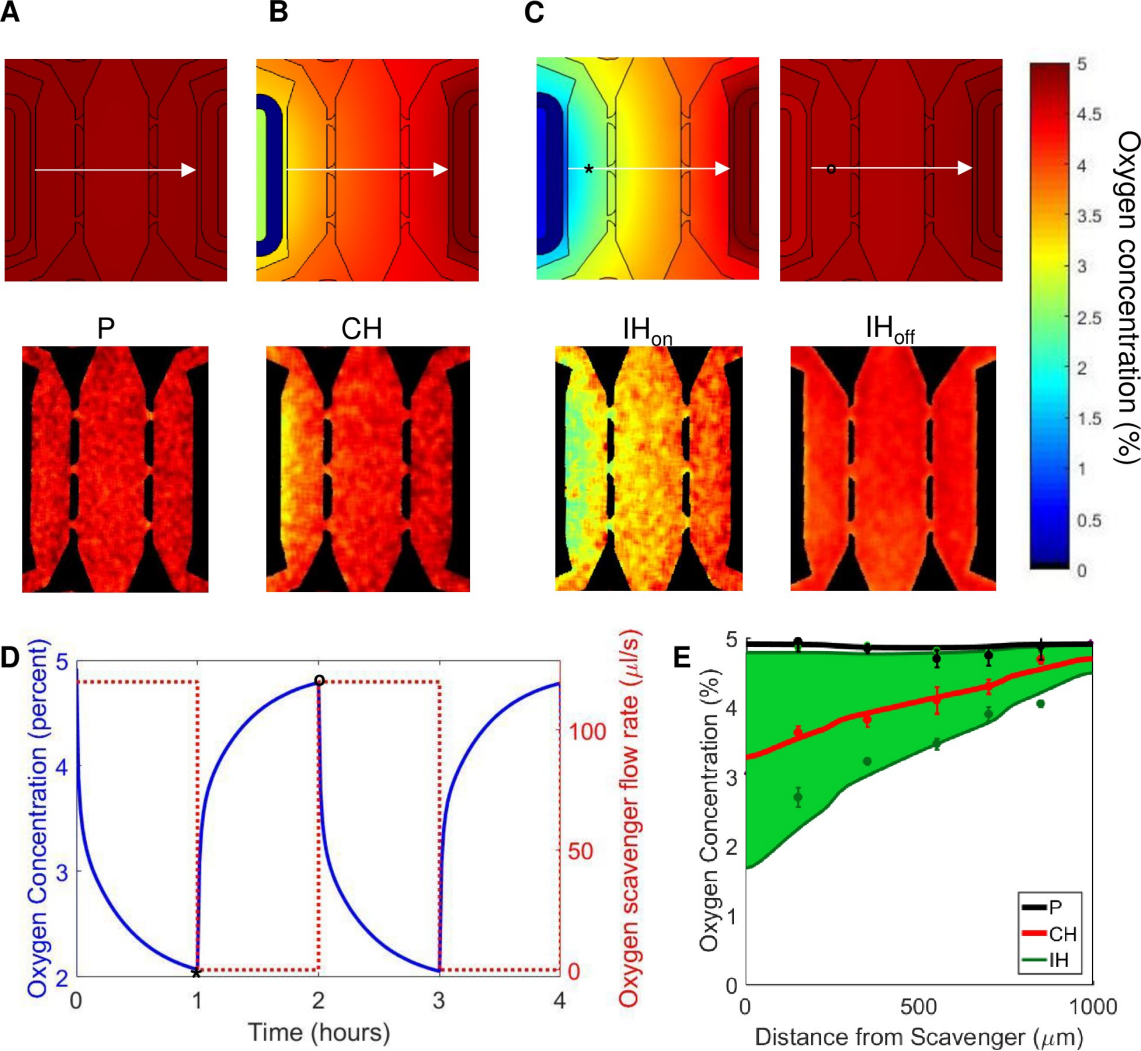
Finite element simulations and measurements of steady state oxygen tension. (A-C, top row) Surface maps of theoretical oxygen tension in different conditions are shown. (A) Physioxia condition (P) with no oxygen scavenger, (B) constant chronic hypoxia condition (CH) with a constant flow of 0.35 M sodium sulfite flowing at 120 μl min^-1^, and (C) intermittent hypoxia (IH) condition of alternating 1 M sodium sulfite flowing at 120 μl min^-1^ for an hour (IH_on,_ left panel) and no flow of oxygen scavenger for an hour (IH_off_, right panel). (A-C, bottom) Experimental oxygen maps were constructed using a PhLIM technique for the P (A, bottom), CH (B, bottom), and IH (C, bottom) conditions. The IH_on_ measurements were taken an hour after flowing sodium sulfite, and the IH_off_ measurements were taken an hour after stopping flow. (D) Temporal variations of the oxygen tension at a point in the left stromal chamber from the COMSOL model in (C, top). The asterisks (*) and circle (o) on the oxygen maps corresponds to the low and high points of the graph, respectively. (E) Comparing the oxygen profiles of all the varying conditions along the central line of the three chambers (arrow in A-C). The conditions are P (black), CH (red), IH (green). The IH case is represented by a green area to illustrate the range of oxygen profiles between the two extreme states of the condition. The solid lines are values from the COMSOL model, and the points are averages from the PhLIM measurements for the three conditions (n = 3 for each conditions).

Each experimental condition was modeled using a 3D simulation, and 2D projections are shown from a slice of the model in the xy plane 50 μm from the bottom of the device (plane at vertical center). In addition, a horizontal line (represented by a white arrow) was drawn in the middle of the chambers to quantify the gradient across the device (Fig 3A–3C, top row). With no scavenger present (P condition) the oxygen concentration remains constant near 5% O_2_ throughout the device (Fig 3A, top, and Fig 3E). Although the device is fairly symmetric, the placement of the media lines on the left side of the central chamber (Fig 1A) raises oxygen tension slightly in the left stromal chamber. The CH condition (0.7 x 10^−6^ mol s^-1^ sodium sulfite) created a steady state oxygen gradient from 3.2% O_2_ in the left chamber to 4.6% O_2_ in the right chamber (Fig 3B, top, and Fig 3E), and a mean concentration of 4% O_2_ in the central chamber.

The IH condition was designed to have the same average oxygen concentration over time as CH. After one hour of 2 x 10^−6^ mol s^-1^ sodium sulfite flowing through the scavenger line in the IH condition, the oxygen tension ranged from 1.7% (left) to 4.5% (right) O_2_ (Fig 3C, top left, and Fig 3E). After one hour of no scavenger flow, the oxygen profile resembled the physioxia case with the average concentration being only slightly less than 5% O_2_ (Fig 3C, top right, and Fig 3E). The time averaged mean concentration in the central chamber over a complete cycle was 4% O_2_, similar to that of CH. The temporal variations of a fixed position in the middle of the left chamber is shown by the * and **o** symbols in Fig 3C to demonstrate the dynamics in our device (Fig 3D). This figure also shows the step function of the flow of oxygen scavenger going from 120 μl min^-1^ to no flow. Although the sodium sulfite reacts with oxygen quickly to lower the concentration in the scavenger channel, there is a lag to reach the final oxygen concentration in the tissue chambers (Fig 3D). In these experiments, we used molar concentrations of sodium sulfite to ensure a zero concentration of oxygen within the solution. The reaction of sodium sulfite depends only on the concentration of sodium sulfite and is zero order with respect to oxygen [25]. The kinetic equation of the process indicates that an aqueous solution of 0.35 M sodium sulfite quickly (minutes) depletes all the oxygen before entering the microfluidic device. Thus, the sodium sulfite solution creates a near zero boundary condition at the scavenger channel, and serves as an oxygen sink. The scavenger depletes the oxygen in the tissue chambers progressively impacting the regions away from the scavenger line. This process achieves a steady state within approximately one hour.

Experimental oxygen measurements were taken once the dye reached equilibrium within the device. The oxygen maps of the experimental conditions are shown in Fig 3A–3C, bottom, with the measurements for the intermittent hypoxia condition taken one hour after each cycle began. The experimental oxygen concentrations (mean top to bottom for each location from the scavenger) for each condition (Fig 3E) were in agreement with the simulated values (Fig 3A–3C, top row).

**Fig 4:**
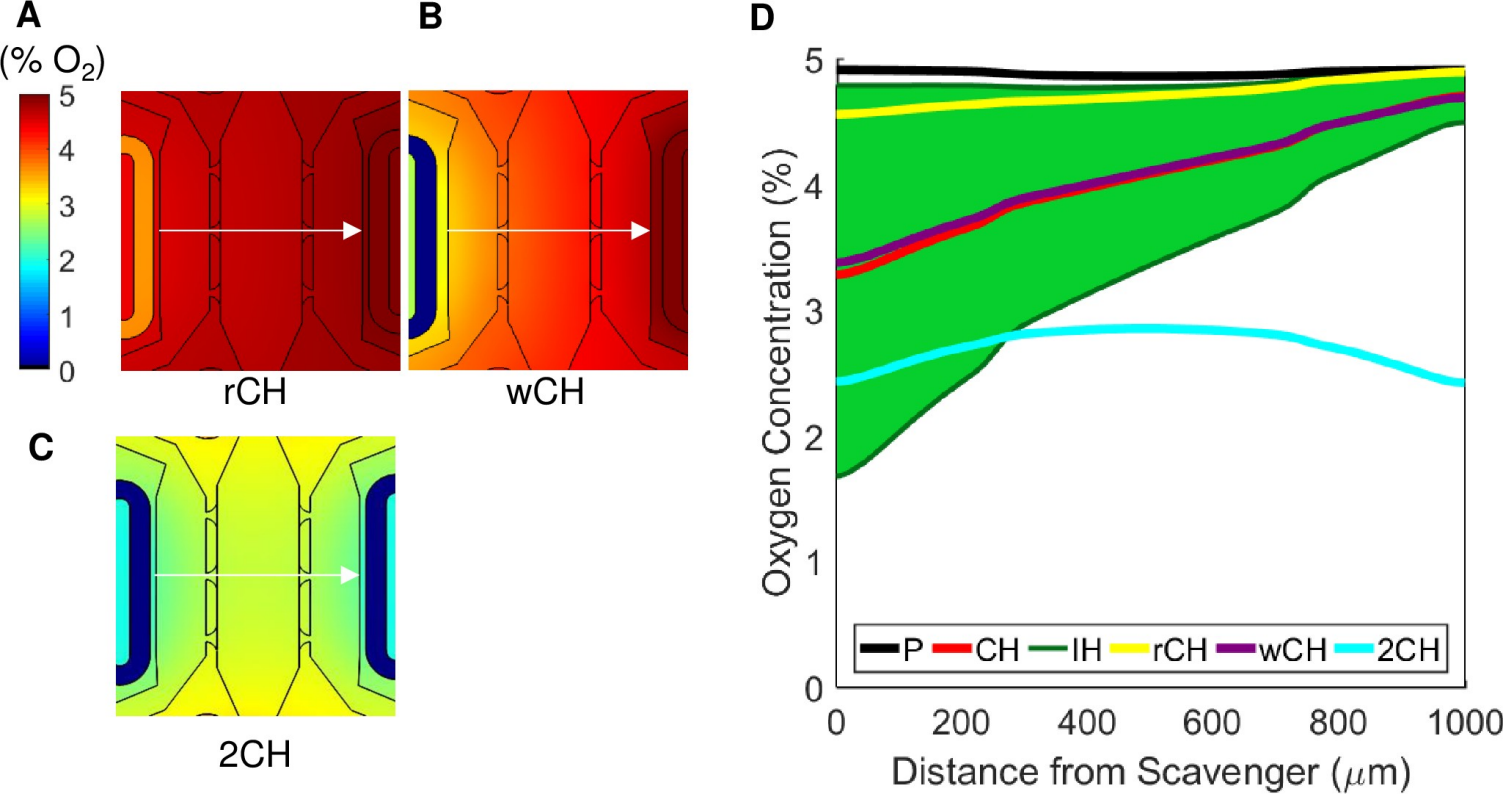
Varying device parameters. (A) Reduced mass flow rate condition (rCH) with 0.07 x 10^−6^ mol s^-1^ of sodium sulfite. (B) Increased wall distance (wCH) between the scavenger channel and stromal chamber to 60 μm. (C) Two active scavenger lines (2CH). (D) Comparing the oxygen profiles of all the varying conditions along the central line of the three chambers (arrow in A-C) with the experimental conditions from Fig 3. The conditions are P (black), CH (red), IH (green), rCH (yellow), wCH (purple), and 2CH (blue).

To illustrate the utility of the microfluidic device for different spatial and temporal oxygen gradients, we performed three additional simulations (Table 2, Fig 4). The first condition, rCH, consisted of reducing the mass flow rate of sodium sulfite to 1/10^th^ the original speed compared to CH (0.07 mol s^-1^, Fig 4A) which increases the oxygen concentration (range of 4.5% to 4.9% O_2_). Next, the condition wCH manipulated the oxygen profile by changing the thickness of the PDMS wall that separates the tissue chambers and the scavenger line. By doubling this wall to 60 μm compared to CH (wall thickness of 30 μm), and using the original mass flow rate of 0.7 x 10^−6^ mol s^-1^, the overall concentration of oxygen was also increased (range of 3.4% to 4.7% O_2_, Fig 4B). Lastly, the condition 2CH drastically changed the oxygen profile by flowing 0.7 x 10^−6^ mol s^-1^ sodium sulfite through both scavenger lines. This condition lowered the overall concentration of oxygen in the device by scavenging both sides. In contrast to all previous simulations, the middle chamber had the highest oxygen concentration as it was the furthest from the scavenger lines. The oxygen tension range in this case was 2.4% to 2.8% O_2_ (Fig 4C). The oxygen quantification of each condition is shown in Fig 4D and represents the concentration of oxygen at each distance along the middle of the chamber (arrow in Fig 4A–4C). As with the previous simulations, because of the subtle asymmetry of the device, the oxygen gradient is not perfectly symmetric although the two scavenger lines have the same mass flow rate of sodium sulfite. The simulations show a range of oxygen tensions that can be achieved by varying parameters such as oxygen scavenger concentration, time exposed to an oxygen scavenger, flow rate of the oxygen scavenger, membrane wall thickness, and number of active scavenger lines.

### Angiogenesis within the three-chambered device

The ECFC-ECs and NHLFs in the central chamber were allowed to grow in a fibrin gel independently for the first four days of the experiment until early vasculature structures were apparent. On day 4, NHLFs in a fibrin gel were loaded into the adjacent left and right chambers, and after 24 hours of incubation, the NHLFs began to spread and resume normal fibroblast morphology. Sprouting vasculature into the left and right stromal chambers was apparent after 6 days.

**Fig 5:**
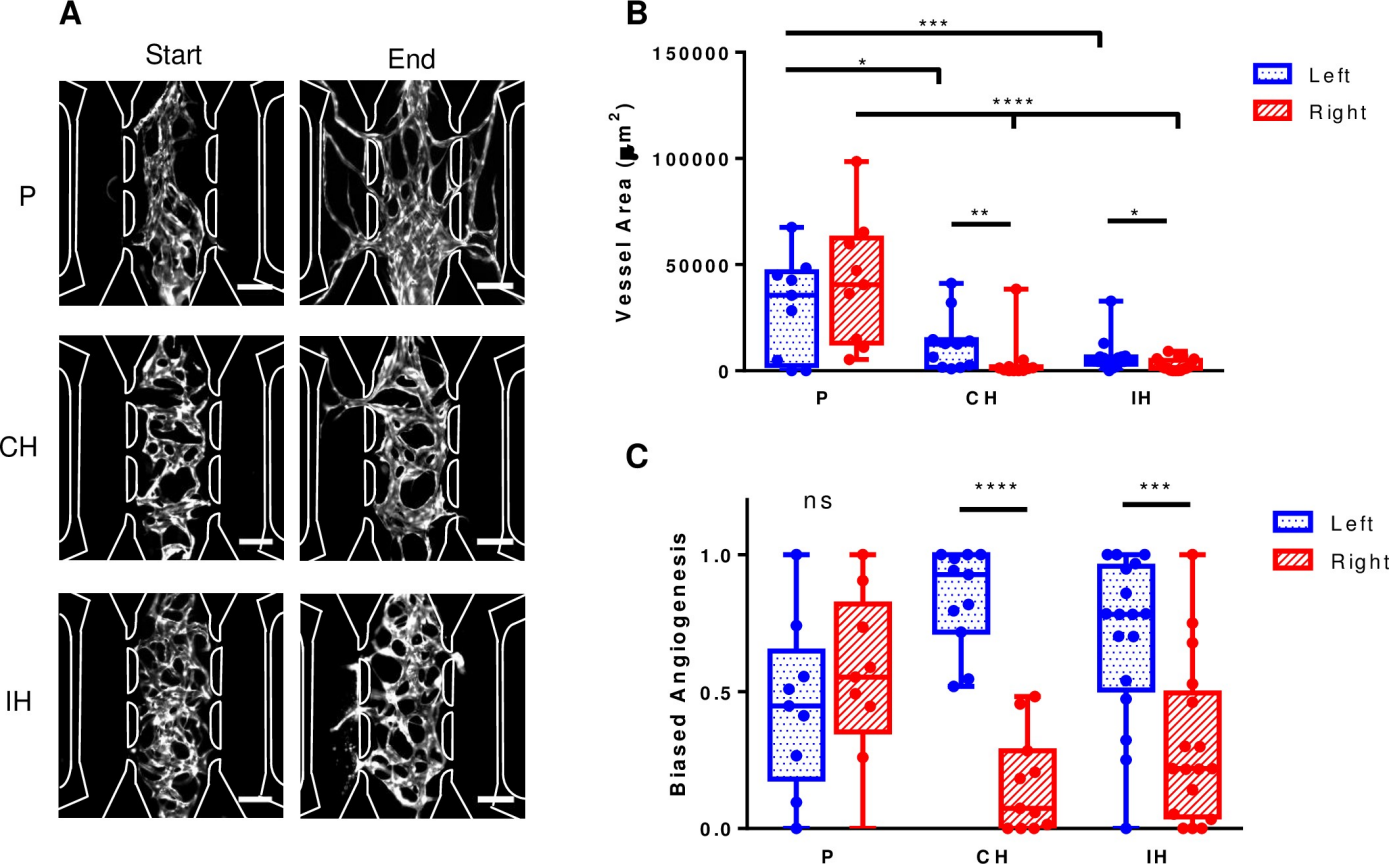
Biased angiogenesis due to hypoxia. (A) Representative images from the P (top), CH (middle), and IH (bottom) conditions. In each category, images of the vascular network before (left) and after (right) the condition was applied is shown. (B) The total vessel area for each category in the left and right stromal chambers were measured and compared. For both hypoxic conditions, the vessel area in the left stromal chamber (closer to the scavenger channel) was significantly higher than the right chamber. (C) Biased angiogenesis was calculated for the left and right stromal chambers in each condition and compared to each other. Both hypoxic conditions had significantly more bias in the left stromal chamber. P, n = 8; CH, n = 11; IH, n = 17. Scale bar = 200μm * p < .05, ** p < .005, *** p < .0005, **** p < .0001.

At the end of the experiment, each condition had devices that sprouted vessels into the adjacent chambers. To confirm the functionality of the vessel network, nonfluorescent endothelial and fibroblast cells were grown in the microfluidic devices under experimental conditions. FITC-dextran was then introduced through the media lines and it then flows throughout the vasculature which forms anastomoses with the fluidic lines (S2 Fig). Fig 5A shows representative images of the three conditions at the start (left panels) and end (right panels) of the experiment. After images of the left and right chambers were randomized and blinded for image analysis, parameters of vessel area and angiogenesis bias were calculated. In the P condition, the left chamber had an average area of 35,577 (8,034–46,742) μm^2^ and the right chamber had an average area of 40,582 (10,109–62,537) μm^2^. This generated values for angiogenesis bias of 0.45 (0.18–0.65) and 0.55 (0.35–0.82) for the left and right chambers, respectively, which were not statistically significant. In the CH condition, the left chamber had an average area of 12,606 (1,644–14,696) μm^2^, which was significantly higher than the right chamber with an average area of 1,187 (167–2767) μm^2^_,_ p < 0.05. This result was also present in the angiogenesis bias for the left and right chambers which were 0.93 (0.72–1.0) and 0.07 (0.0–0.28) respectively (p < .0001). Lastly, the IH case also had significantly different vessel areas in the adjacent chambers with the left chamber having an average of 4,664 (3,121–6539) μm^2^ and the right chamber having an average area of 1,289 (300–4970) μm^2^, p < 0.05 . This trend produced an angiogenesis bias of 0.78 (0.51–0.96) and 0.22 (0.04–0.49) for the left and right chambers which was statistically significant (p < .0005) (Fig 5B and 5C).

We also created control conditions that contained no fibroblasts in the adjacent compartments (plain fibrin) under physioxia and constant chronic hypoxic conditions. In the physioxia case, there were no angiogenic sprouts into the adjacent chambers. On the other hand, the device with plain fibrin in the adjacent chambers had degraded vessels under the constant chronic hypoxic condition (data not shown).

## Discussion

A promising use of organ-on-a-chip technology is the ability to mimic and test disease states. Hypoxia is a prominent feature of diseases such as wound healing, ischemia, and cancer. Hence, the ability to control oxygen within a microfluidic device provides a unique opportunity to understand the impact of oxygen tension at high spatial and temporal resolution. While previous studies may set an incubator to a specific oxygen concentration, this does not create the oxygen concentration gradients that are relevant to human physiology. The structure of the microcirculation is sensitive to oxygen tension, and our device design demonstrates the ability to spatially (microns) and temporally (minutes) control the oxygen concentration around a steady vascular network to study these effects. Our device can introduce various combinations of cells and extracellular matrix at different time points as well as manipulate the oxygen concentration to simultaneously compare the effects of a physioxic or hypoxic stromal chamber on a neighboring vascular network. While the oxygen concentrations and gradients we used represent conditions, such as the earlier stages of cancer, a larger oxygen tension gradient might be necessary for studying other diseases or more advanced cancer. Because our oxygen scavenger is not directly interacting with our tissue chambers, 0% oxygen tension cannot be achieved in the tissue chambers. However, lower oxygen tensions can be achieved by lowering the ambient concentration in the incubator or changing the thickness of the PDMS layer separating the scavenger from the tissue. The steepest concentration gradient achieved in the current design was about 2%^.^mm^-1^. To further increase the gradient, the distance between scavenger and the physioxia line can be further decreased by reducing the tissue chamber sizes.

Our ability to experimentally measure oxygen within the device provides the opportunity to validate the performance and accuracy of the platform and model simulations. PhLIM provides high spatial resolution in 3D that is capable of capturing an entire oxygen map. This is a distinct advantage over traditional oxygen sensors and probes that are placed on one surface or in a single position and rely on models to extrapolate the 3D oxygen map. The PhLIM oxygen measurements closely match the oxygen values predicted by the model over a range of experimental conditions.

Additionally, through the use of COMSOL modeling we demonstrate that the oxygen profile in the device can be controlled and fine-tuned by using easily adaptable parameters, such as mass flow rate, thickness of the PDMS wall, and the number of scavenger lines. Our simulations demonstrate that each parameter affects the oxygen tension profile in different ways. The rCH and 2CH conditions drastically changed the oxygen gradient; whereas wCH has a subtle impact.

While other intermittent hypoxia studies typically switch between 20% and 1% O_2_ [14–16], it is physiologically more relevant to cycle between 5% and 1% O_2_ to replicate disease states such as the tumor microenvironment in the presence of a leaky vasculature [3]. In our simulations and experiments, we are able to easily produce oxygen values in the 1–5% O_2_ range. Although we only tested one frequency in this study, our device has the ability to recreate longer or more extreme hypoxic frequencies. While other studies have found results that may suggest that intermittent hypoxia can increase proliferation or migration of fibroblasts, these studies often cycle between 20% O_2_ and 1% O_2_ [34]. Even though we did not see these results in our intermittent hypoxia condition, we believe that our results are more relevant to human physiology because we chose to cycle between oxygen concentrations that are apparent *in vivo*.

To demonstrate the biological relevance of our device, we investigated the ability of constant chronic and intermittent hypoxia to stimulate angiogenesis. In physioxia (5% O_2_), vessel growth was relatively uniform into both adjacent tissue compartments. Although not statistically different, small variances between the left and right compartments could be due to the slight asymmetry in the design. Our device was able to recreate sprouting angiogenesis into a hypoxic tissue microenvironment under both constant chronic and intermittent conditions. This observation is consistent with a normal biological response [1,2,34]. The hypoxic conditions generated less total vasculature than the physioxia case. This can be explained by the lower mean oxygen tension within the vasculature chamber of the CH and IH cases (as low as 3.5% and 2.5%, respectively, compared to 5%). The lower oxygen tension in the two hypoxic conditions likely results in hypoxic stress to the vascular network and reduced overall sprouting.

The constant chronic hypoxia condition had more significant angiogenesis bias towards the low oxygen chamber compared to the intermittent hypoxia condition as well as greater vessel area. This is probably due to the range of oxygen concentrations we’ve chosen as well as frequency of the cycle. Alternatively, as the constant chronic hypoxia case is a continuous exposure to hypoxia, the stressed fibroblasts in the left chamber might have continually expressed pro-angiogenesis factors to encourage more vessel growth relative to the intermittent hypoxia condition. As shown previously by our group, the frequency of the intermittent hypoxia cycles can change how a vessel responds, which can be correlated to the amount of vascular endothelial growth factor that is secreted by hypoxic fibroblasts [35].

## Conclusion

The results of this study show the effectiveness of microfluidic devices to control and manipulate oxygen tension at high spatial and temporal resolution. The device design demonstrates the ability to load different types of cells at different time points, which provides the opportunity to model many different normal and pathological states. We chose 3D sprouting angiogenesis to demonstrate the biological relevance of the device design, and we were able to replicate the well-described angiogenic response of hypoxic (constant chronic and intermittent) fibroblasts. The simple and flexible design of the devices provides an opportunity to enhance understanding of important disease processes present in cancer, ischemic heart disease, and wound healing.

## Supporting information

S1 Fig Reaction rate of sodium sulfite does not vary significantly with temperature. Varying concentrations of sodium sulfite were prepared in a 96 well plate. The concentration of O_2_ was measured over time and the rate of change of O_2_ was calculated by assuming a linear drop in concentration from 21% O_2_ at t = 0. The rate of influx of oxygen was assumed constant with respect to the temperature.
(PPTX)

S2 Fig Permeability of vessel network. FITC-dextran was introduced in the fluidic lines and enters the vasculature through anastomoses with the fluidic lines of the device. Scale bar = 200μm.
(PPTX)
