# Supplementary figures and images for: Microfluidic device to attain high spatial and temporal control of oxygen
Source: PLoS One. 2018 Dec 20;13(12):e0209574. doi: 10.1371/journal.pone.0209574 (PMC6301786; doi:10.1371/journal.pone.0209574)

## Slide 1
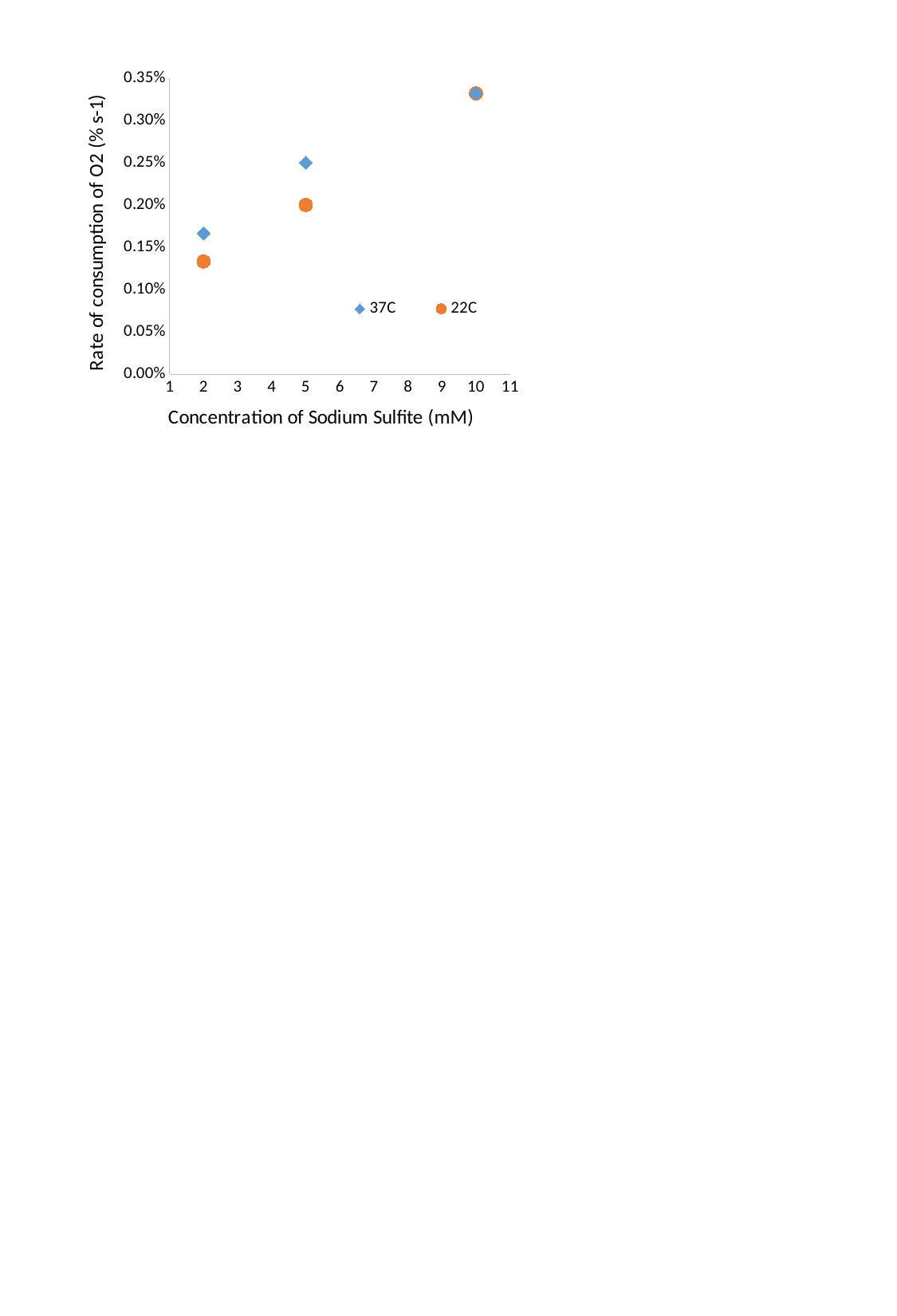

### Chart
| Category | 37C | 22C |
|---|---|---|

Supplement: S1 Fig — Varying concentrations of sodium sulfite were prepared in a 96 well plate. The concentration of O2 was measured over time and the rate of change of O2 was calculated by assuming a linear drop in concentration from 21% O2 at t = 0. The rate of influx of oxygen was assumed constant with respect to the temperature. (PPTX) [file pone.0209574.s001.pptx]

## Slide 1
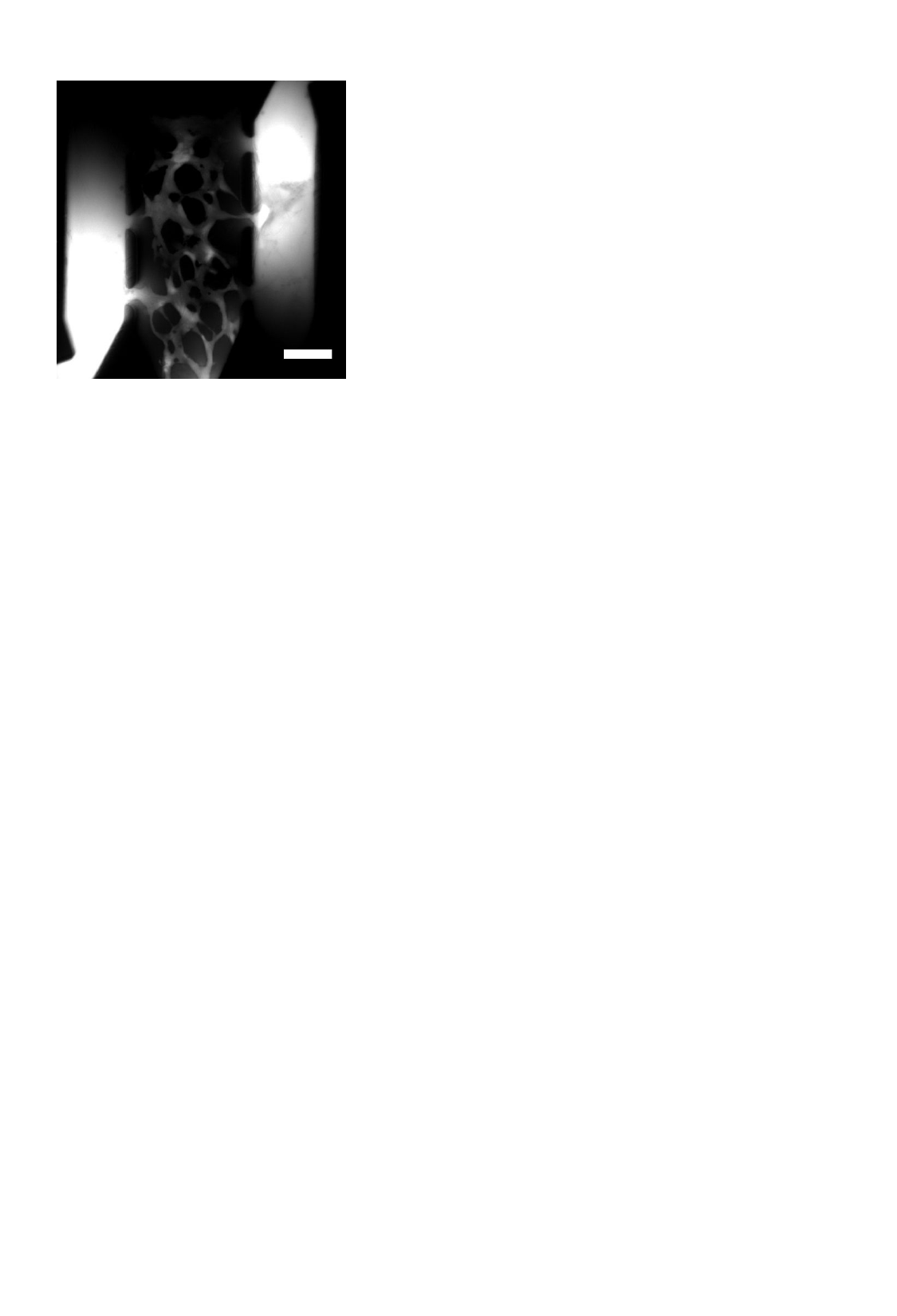

Supplement: S2 Fig — FITC-dextran was introduced in the fluidic lines and enters the vasculature through anastomoses with the fluidic lines of the device. Scale bar = 200μm. (PPTX) [file pone.0209574.s002.pptx]
